# Clinical Characteristics and Outcomes of Breast Cancer in Botswana

**DOI:** 10.1200/GO.22.41000

**Published:** 2022-05-05

**Authors:** James Wester, Isaac Nkele, Bosa Motladiile, Neo Tapela, Joe Makhema, Shahin Lockman, Scott Dryden-Peterson

**Affiliations:** ^1^ Northwestern University Feinberg School of Medicine, Chicago, IL; ^2^ Botswana Harvard Partnership, Gaborone, Botswana; ^3^ Brigham and Women's Hospital, Boston, MA

## Abstract

Breast cancer is the leading cause of cancer death in women worldwide. In sub-Saharan Africa, breast cancer has a lower incidence than high-resource settings like the United States, but the mortality-to-incidence ratio is significantly higher in sub-Saharan Africa. Limited primary literature regarding the clinical characteristics of breast cancer is available in many regions of sub-Saharan Africa. In this report, we aim to characterize the clinical attributes and outcomes of women with breast cancer in Botswana.

**METHODS:**

The prospective Thabatse cancer cohort in Botswana began enrolling participants (18 years or older, biopsy-confirmed cancer) in 2010 and recruited participants from the four main oncology referral centers in Botswana. Baseline clinical data was obtained, and participants were followed quarterly for 5 years. Certification of death was obtained from families, providers, and death certificates.

**RESULTS:**

Eight hundred three women with breast cancer in Botswana, enrolled between 2010 and the end of December 2020, were included in this analysis. Average age at enrollment was 53 years old. On immunohistochemistry, 331 (41.2%) of tumors were hormone positive, 57 (7.1%) were HER2+, 114 (14.2%) were Triple-Negative, and 301 (37.5%) were unknown. At diagnosis, 23 (2.9%) of participants had stage I disease, 163 (20.3%) stage II, 343 (42.7%) stage III, 80 (10.0%) stage IV, and 194 (24.2%) unknown. 515 (64.1%) of participants had surgery; of those who had surgery, 54 (10.5%) had a lumpectomy, 459 (89.1%) had a mastectomy, and 2 (0.4%) had a self-amputation. 458 (57.0%) participants received chemotherapy, and 277 (34.5%) received radiation therapy. 5-Year survival, calculated from enrollment date, was 47.7% (95% CI 43.7 to 52.1).

**CONCLUSION:**

This study demonstrates the unique burden of breast cancer disease in Botswana utilizing an ongoing longitudinal prospective study. Participants in our study on average presented at late stage, received invasive surgery, and had limited access to chemotherapy and radiation therapy.

